# Cardiopulmonary Resuscitation in Interfacility Transport: An International Report Using the Ground Air Medical Quality in Transport (GAMUT) Database

**DOI:** 10.1155/2020/4647958

**Published:** 2020-07-10

**Authors:** Utpal S. Bhalala, Neeraj Srivastava, M. David Gothard, Michael T. Bigham

**Affiliations:** ^1^The Children's Hospital of San Antonio, San Antonio, TX, USA; ^2^Baylor College of Medicine, Houston, TX, USA; ^3^Biostats Inc., East Canton, OH, USA; ^4^Akron Children's Hospital, Akron, OH, USA

## Abstract

**Background:**

With the regionalization of specialty care, there is an increasing need for interfacility transport from local to regional hospitals. There are very limited data on rates of cardiopulmonary resuscitation (CPR) during medical transport and relationship between transport-specific factors, such as transport program type and need of CPR during transport of critically ill patients. We present the first, multicenter, international report of CPR during medical transport using the large Ground and Air Medical qUality Transport (GAMUT) database.

**Methods:**

We retrospectively reviewed the GAMUT database from January 2014 to March 2017 for CPR during transport. We determined the overall CPR rate and CPR rates for adult, pediatric, and neonatal transport programs. The rate of CPR per total transports was expressed as percentage, and then, Spearman's rho nonparametric associations were determined between CPR and other quality metrics tracked in the GAMUT database. Examples include advanced airway presence, waveform capnography usage, average mobilization time from the start of referral until en route, 1^st^ attempt intubation success rate, and DASH1A intubation success (definitive airway sans hypoxia/hypotension on 1^st^ attempt). Data were analyzed using chi-square tests, and in the presence of overall significance, post hoc Bonferroni adjusted *z* tests were performed.

**Results:**

There were 72 programs that had at least one CPR event during the study period. The overall CPR rate was 0.42% (777 CPR episodes/184,272 patient contacts) from 115 programs reporting transport volume and CPR events from the GAMUT database during the study period. Adult, pediatric, and neonatal transport programs (*n* = 57, 40 and 16, respectively) had significantly different CPR rates (*P* < 0.001) i.e., 0.68% (555/82,094), 0.18% (138/76,430), and 0.33% (73/21,823), respectively. Presence of an advanced airway and mobilization time was significantly associated with CPR episodes (*P* < 0.001) (Rs = +0.41 and Rs  = −0.60, respectively). Other transport quality metrics such as waveform capnography, first attempt intubation, and DASH1A success rate were not significantly associated with CPR episodes.

**Conclusion:**

The overall CPR rate during medical transport is 0.42%. Adult, pediatric, and neonatal program types have significantly different overall rates of CPR. Presence of advanced airway and mobilization time had an association with the rate of CPR during transport.

## 1. Introduction

Regionalization of healthcare has proven to improve outcomes through concentrating the resources and expertise for sick and injured patients in tertiary and quaternary hospitals [1]. As a result, over 500,000 air transports and many-fold more ground critical care transports occur annually in the United States alone [2–4]. Some of these patients require critical care interventions including endotracheal intubation and the most extreme intervention, cardiopulmonary resuscitation for cardiac arrest. Unfortunately, there is a limited data on rates of cardiopulmonary resuscitation (CPR) during medical transport or the relationship between transport program type and other quality performance measures amongst those patients that need CPR during transport [5]. Ground Air Medical qUality in Transport (GAMUT) Quality Improvement Collaborative includes a large, international database on medical transport quality metrics [6]. Using this database, we sought to determine the rate of cardiopulmonary resuscitation (CPR) during medical transport and relationship between transport-specific factors.

Though the survival to hospital discharge after out-of-hospital cardiac arrest (OHCA) has improved from 8.2% to 10.4%, it remains dismal as compared to the survival after in-hospital cardiac arrest (IHCA) [7]. The factors, which determine the outcomes of the OHCA, are circumstances around the cardiac arrest, estimated downtime, and quality of cardiopulmonary resuscitation (CPR) [7–9]. The goal of medical transport by emergency medical service teams is to improve the clinical status in a critically ill patient who is distant from a hospital capable of providing a requisite level of critical care. This is feasible as the critical care transport service functions as an extension of the intensive care unit (ICU), capable of providing tertiary-level critical care throughout its referral region. With an increasing number of complex critically ill and injured patients transported for a higher level of care, it is likely that a growing proportion of these patients will need CPR during transport.

Faced with increasing transport populations and improving science related to CPR quality and outcomes, we sought to initially describe the scope of CPR during medical transport among our cohort of patients. In doing so, we further sought to understand characteristics of programs with higher CPR rates and any associations of program-level performance for other quality metrics.

## 2. Methods

This retrospective study was conducted using the international, multicenter GAMUT database [10–12]. Study data were captured and managed using research electronic data capture (REDCap) hosted at the GAMUT QI collaborative [13]. The GAMUT database was then assessed retrospectively over the study period, January 2014–March 2017, for reported CPR during transport. Monthly institutional GAMUT data were aggregated from January 2014 to March 2017 by institution. To eliminate spurious associations, institutions with <20 transports were excluded. GAMUT defines transport CPR as the number of transports during which CPR is performed from the time the transport team assumes partial or complete care until the patient hand-off is completed at the destination facility and accounts for multiple episodes of chest compressions in a single transport as only one CPR episode. CPR events in progress when the team arrives are excluded for the purposes of this study. The rate of CPR per total transports was expressed as a percentage. We determined the overall CPR rate and CPR rate for different types of transport programs. We defined transport programs as follows: (1) adult transport program has >50% of patient contacts with age ≥18 yrs, (2) pediatric transport program has >50% pediatric patient contacts with majority of pediatric contacts with age between 29 days and <18 yrs, and (3) neonatal transport program has >50% pediatric patient contacts with majority of pediatric contacts with age between 0 and 28 days. GAMUT data were imported into SPSSv24.0 software for analysis purposes (IBM Corporation; Armonk, NY). For each institution, every month of data from January 2014 to March 2017 containing both total transport contacts and number of runs with CPR GAMUT data elements were aggregated into a collective summary. The rate of CPR was then determined across all institutions as the total number of runs with CPR divided by total transport contacts. A comparison of these rates by program and type (adult, pediatric, and neonate) was performed via the Pearson chi-square test with post hoc Bonferroni adjusted *z* tests. CPR rates were presented by program type and institutional outliers determined via 1.5^*∗*^IQR rule. Outliers were identified for further institutionally specific study to determine potential influences on the rate of CPR. Additional analyses explored the relationship between metrics already captured in the GAMUT database to rates of CPR. Spearman's rho nonparametric associations were determined due to the presence of influential outliers between CPR and advanced airway (per total transports), waveform capnography usage (per total advanced airways), average mobilization time in minutes from the start of referral until en route, 1^st^ attempt intubation success rate (per total intubation attempts), and DASH1A intubation success (definitive airway sans hypoxia/hypotension on 1^st^ attempt per total intubation attempts).

## 3. Results

There were 73 institutions that had at least one CPR event during the study period. The overall CPR rate was 0.42% (777 CPR episodes/184,272 patient contacts) from 115 institutions reporting transport volume and CPR events from the GAMUT database during the study period. Adult transport programs (*n* = 57) had a CPR rate of 0.68% (555/82,094) (Table 1). Pediatric transport programs (*n* = 40) had a CPR rate of 0.18% (138/76,430). Neonatal transport programs (*n* = 16) had a CPR rate of 0.33% (73/21,823). All three transport program types had a different overall rate of CPR (*P* < 0.001 via the chi-square test and post hoc Bonferroni adjusted *z* tests). Additionally, there were (*n* = 2) program types with undefined patient transport subgroup data with a CPR rate of 0.28% (11/3,925). After adjustment for patient transport type, there were 5 institutions with a rate of CPR over 1.5^*∗*^IQR higher (worse) than their peers.

Two transport quality metrics were significantly associated with CPR: presence of an advanced airway and mobilization time (*P* < 0.001) (Rs = +0.41 and Rs = −0.60, respectively). Other transport quality metrics such as waveform capnography, first attempt intubation, and DASH1A success rate were not significantly associated with CPR.

## 4. Discussion

This is the first report describing rates of CPR during ground and air medical transport and association of transport quality metrics with CPR using a large, international database. The results suggest overall very low CPR rate during medical transport. The findings suggest that critically ill patients are less commonly transported with an on-going CPR. In fact, amongst pediatric transport programs surveyed, Noje et al. reported that only 32% of programs would transport with active CPR in progress [14]. Adult critical care transport programs more commonly span the spectrum of EMS and critical care transport, and thus may more commonly transport patients undergoing active CPR, particularly those retrieved during scene transports. The results may be markedly different between programs that perform scene runs vs interfacility missions. Programs responding to the scene may be more likely to continue CPR in transit and will be less likely to perform termination of resuscitation (or choose not to initiate resuscitation) then those only performing interfacility transport.

Studies have shown improved outcomes of in-hospital cardiac arrest (IHCA); i.e.,the survival to hospital discharge after IHCA has improved from 14.3% in 2000 to 43.4% in 2009 [15]. However, survival to hospital discharge after out-of-hospital cardiac arrest (OHCA) remains only 8.2–10.4% [7,16]. There is a scarcity of literature around OHCA and CPR during medical transport. One could speculate that transport-related CPR events may align more with in-hospital outcomes, as the resuscitative team and clinical expertise are alongside the patient at the time of cardiac arrest. EMS-witnessed cardiac arrest survival rates of 16–18% are ahead of OCHA survival to discharge [17,18]. The findings of higher CPR rates in adult transport as compared to pediatric transports are probably related to overall higher risk of cardiac arrest in adults due to comorbidities related to aging. We believe that findings of very low CPR rates during medical transport in children in our study are related to several factors. First, the overall rate of OHCA in children is low (2.28–9 per 100, 000 person-years) [19,20]; second, most pediatric OHCA are caused by progressively worsening and/or persistent hypoxemia and/or hypotension, whereas most adult OHCA are related to potentially reversible, primary cardiac event. Due to the underlying pathophysiology of cardiac arrest, less physiologic reserve, and high metabolic demand, children are more likely to progress to anoxic brain injury and death [21–23]; third, tendency to perform CPR in the emergency room rather than transporting the children with an ongoing CPR. Due to survival benefits, American Heart Association (AHA) guidelines recommend transferring the adult patients with OHCA to nearest facility capable of percutaneous transcoronary angioplasty (PTCA) [24]. Based on these recommendations, transport teams are more likely to transport adult victims of OHCA to the nearest PTCA center with an ongoing CPR.

The goal of patient transport by emergency medical service teams is to improve the clinical status in a critically ill patient who is distant from a hospital capable of providing a requisite level of intensive care. An increase in interfacility medical transport has created practice variations. Practice variations among teams transporting patients are largely attributable to lack of progress in establishing medical transport performance metrics. Such metrics are essential for benchmarking high performing transport-specific operations to establish critical care best practices [12,25,26]. Using the GAMUT database, our study focused on evaluating transport quality metrics in those patients who needed CPR during medical transport. Our report showed an association between presence of an advanced airway and occurrence of CPR during medical transport. This suggested that either patient with presence of an advanced airway during medical transport were much sicker than those who did not have an advanced airway during transport and that these patients progressed to OHCA and needed CPR during medical transport. The other possibility was that an advanced airway was placed during or around CPR in order to support airway and breathing. It is also possible that the advanced airway may have been harmful, and there may have been an epiphenomenon of peri-intubation arrest. The evidence around advanced airway management during CPR is conflicting. In pediatric OHCA, prehospital advanced airway management (AAM) was not associated with an increased chance of neurologically favorable survival compared with bag-valve-mask-(BVM-) only ventilation [27]. In another study, among adult patients with OHCA, any type of AAM was independently associated with decreased odds of neurologically favorable survival compared with conventional BVM-only ventilation [28]. In a study through the Cardiac Arrest Registry to Enhance Survival (CARES) database, survival was higher among OHCA receiving endotracheal intubation (ETI) than those receiving supraglottic airway (SGA) and for patients who received no advanced airway than those receiving ETI or SGA [29].

In our study, there was a negative association between mobilization time (MT) and occurrence of CPR during medical transport. MT is defined as the “average time in minutes from the start of the referral phone call to the transport team to the time is en route to the referral facility.” A study by Bigham et al. showed that neonatal and pediatric transports had a longer MT as compared to adult transports. The same study also showed some association with increased MT and unplanned device removal and hypoxic event rates [30]. Our findings of negative association between MT and occurrence of CPR are in alignment with findings of Bigham et al. An association between MT and rate of CPR during transport suggests that the urgency associated with the sickest patients was associated with a short average mobilization time.

Waveform capnography helps with confirming and monitoring advanced airway and breathing in a patient. It also assists with recognition of cardiac arrest and monitoring of quality of CPR [31]. According to 2015 American Heart Association (AHA) guidelines on CPR, end-tidal CO2 (ETCO2) is an important determinant of high-quality CPR [32,33]. In our study, waveform capnography was not associated with occurrence of CPR during medical transportation. This finding suggests that compliance with AHA guidelines on monitoring quality of CPR using waveform capnography during medical transport was poor. The presence of ETCO2 may be largely predicated by the use of an advanced airway (before or after the CPR event). Some EMS services can use ETCO2 captured from bag-mask ventilation or a nasal cannula. It is reasonable to comment that some efforts might be needed educating and encouraging the transport teams to use waveform capnography as a noninvasive modality not only for confirming the patency of airway and appropriate position of an advanced airway during a resuscitative event but also for monitoring quality of CPR during medical transport.

First pass intubation in emergency department has been shown to be associated with relatively small incidence of adverse events including cardiac arrest [34]. Our study did not show any association between first attempt intubation and DASH1A and occurrence of CPR during medical transport. This could be related to overall very low rate of CPR during medical transport and a higher proportion of patients being supported on BVM during a resuscitative event during medical transport. DASH1A would also not be associated with CPR if all the airways were placed after CPR began. A study in Korea revealed that failed initial intubation attempt (FIIA) during OHCA in adult patients was associated with an average delay of 3 min in the time to return of spontaneous circulation (ROSC). In the same study, risk regression analysis revealed a significantly slower ROSC rate during the first 15 min in the FIIA group [35].

This study does have some limitations. First, the nature of the high-level quality improvement database does not allow patient-level analysis, which may uncover unique associations, diagnoses, or patient populations at greater risk for CPR. The GAMUT database also does not provide granular information such as whether the airway was placed before or after the arrest. This introduces two different types of bias (spectrum for airways before and indication for airways placed after CPR). The cohort of patients in the GAMUT database represents only a small fraction of critical care transports and is not likely to be a representative sample. The data are entered retrospectively and locally, so accuracy of entered data are not evaluated. Additionally, anticipated and unanticipated CPR, air vs. ground transport CPR rates, quality of CPR, and outcomes of CPR are all important but not measured by this study. Furthermore, discrimination between scene vs. interfacility transport episodes does not exist but could inform a clearer understanding of transport CPR.

## 5. Conclusion

The overall CPR rate during medical transport was found to be 0.42% in our cohort of patients during interfacility transport. The three different transport program types (adult, pediatric, and neonatal) have a significantly different overall rate of CPR during transport of critically ill patients, with adult programs delivering CPR most commonly, neonatal programs less commonly, and pediatric programs least frequently. Presence of advanced airway and mobilization time had an association with rate of CPR during transport. Further studies are needed to understand the true relationship between the rate of CPR and transport quality metrics and ultimately the impact and outcomes of patients receiving CPR during transport.

## Figures and Tables

**Table 1:** Comparison of aggregated CPR rates by program type.

	Total transport contacts	Contacts with CPR (%)	*P* value
Program type			<0.001
Adult	82094	555 (0.68)	
Pediatric	76430	138 (0.18)	
Neonatal	21823	73 (0.33)	

*Note.* Program types were each significantly different via post hoc Bonferroni adjusted *z* tests.

## Data Availability

The data used to support the findings of this study are available from the corresponding author upon request.

## References

[1] Pollack M. M., Patel K. M., Ruttimann U. E. (1996). Prism III. *Critical Care Medicine*.

[2] The association of air medical services fact sheet. Alexandria, VA. https://aams.org/member-services/fact-sheet-faqs/

[3] Agency for Healthcare Research and Quality (2018). *HCUPnet, healthcare cost and utilization project*.

[4] Rosenthal J. L., Hilton J. F., Teufel R. J., Romano P. S., Kaiser S. V., Okumura M. J. (2016). Profiling interfacility transfers for hospitalized pediatric patients. *Hospital Pediatrics*.

[5] Hartke A., Mumma B. E., Mumma B. E., Rittenberger J. C., Callaway C. W., Guyette F. X. (2010). Incidence of rearrest and critical events during prolonged transport of post-cardiac arrest patients. *Resuscitation*.

[6] Lee S. H., Schwartz H. P., Bigham M. T. (2018). From the street to the ICU: a review of pediatric emergency medical services and critical care transport. *Translational Pediatrics*.

[7] Daya M. R., Schmicker R. H., Zive D. M. (2015). Out-of-hospital cardiac arrest survival improving over time: results from the Resuscitation Outcomes Consortium (ROC). *Resuscitation*.

[8] Cheskes S., Schmicker R. H., Rea T. (2017). The association between AHA CPR quality guideline compliance and clinical outcomes from out-of-hospital cardiac arrest. *Resuscitation*.

[9] Martinell L., Nielsen N., Herlitz J. (2017). Early predictors of poor outcome after out-of-hospital cardiac arrest. *Critical Care*.

[10] Collaborative GQ (2016). GAMUT QI Collaborative Consensus Quality Metrics. http://gamutqi.org/GAMUT%20Metricsversion%205.16.2016.pdf.

[11] Schwartz H. P., Bigham M. T., Schoettker P. J. (2015). Quality metrics in neonatal and pediatric critical care transport. *Pediatric Critical Care Medicine*.

[12] Bigham M. T., Schwartz H. P. AMPA Quality Metrics Conference Proceedings.

[13] Harris P. A., Taylor R., Thielke R., Payne J., Gonzalez N., Conde J. G. (2009). Research electronic data capture (REDCap)-A metadata-driven methodology and workflow process for providing translational research informatics support. *Journal of Biomedical Informatics*.

[14] Noje C., Bembea M. M., Nelson McMillan K. L. (2019 Jan). A national survey on interhospital transport of children in cardiac arrest^*∗*^.. *Pediatric Critical Care Medicine*.

[15] Girotra S., Spertus J. A., Li Y., Berg R. A., Nadkarni V. M., Chan P. S. (2013). Survival trends in pediatric in-hospital cardiac arrests. *Circulation: Cardiovascular Quality and Outcomes*.

[16] Jayaram N., McNally B., Tang F., Chan P. S. (2015). Survival after out--of--hospital cardiac arrest in children. *Journal of the American Heart Association*.

[17] Hostler D., Thomas E. G., Emerson S. S. (2010). Increased survival after EMS witnessed cardiac arrest. Observations from the Resuscitation Outcomes Consortium (ROC) Epistry-Cardiac arrest. *Resuscitation*.

[18] Meyer L., Stubbs B., Fahrenbruch C. (2012). Incidence, causes, and survival trends from cardiovascular-related sudden cardiac arrest in children and young adults 0 to 35 Years of age. *Circulation*.

[19] Bardai A., Berdowski J., Van Der Werf C. (2011). Incidence, causes, and outcomes of out-of-hospital cardiac arrest in children. *Journal of the American College of Cardiology*.

[20] Li C.-J., Kung C.-T., Liu B.-M. (2010). Factors associated with sustained return of spontaneous circulation in children after out-of-hospital cardiac arrest of noncardiac origin. *The American Journal of Emergency Medicine*.

[21] Sirbaugh P. E., Pepe P. E., Shook J. E. (1999). A prospective, population-based study of the demographics, epidemiology, management, and outcome of out-of-hospital pediatric cardiopulmonary arrest. *Annals of Emergency Medicine*.

[22] Kuisma M., Maatta T., Repo J. (1998). Cardiac arrests witnessed by EMS personnel in a multitiered system: epidemiology and outcome. *The American Journal of Emergency Medicine*.

[23] O’Connor R. E., Al Ali A. S., Brady W. J. (2015). Part 9. Acute coronary syndromes: 2015 American Heart association guidelines update for cardiopulmonary resuscitation and emergency cardiovascular care. *Circulation*.

[24] Young K. D., Seidel J. S. (1999). Pediatric cardiopulmonary resuscitation: a collective review. *Annals of Emergency Medicine*.

[25] Bigham M. T., Schwartz H. P. (2013). Quality metrics in neonatal and pediatric critical care transport. *Pediatric Critical Care Medicine*.

[26] Schwartz H. P., Bigham M. T., Schoettker P. J. (2015). Quality metrics in neonatal and pediatric critical care transport. *Pediatric Critical Care Medicine*.

[27] Ohashi-Fukuda N., Fukuda T., Doi K., Morimura N. (2017). Effect of prehospital advanced airway management for pediatric out-of-hospital cardiac arrest. *Resuscitation*.

[28] Hasegawa K., Hiraide A., Chang Y., Brown D. F. M. (2013). Association of prehospital advanced airway management with neurologic outcome and survival in patients with out-of-hospital cardiac arrest. *JAMA*.

[29] McMullan J., Gerecht R., Bonomo J. (2014). Airway management and out-of-hospital cardiac arrest outcome in the CARES registry. *Resuscitation*.

[30] Bigham M. T., Parrish P. R., Gothard M. D. (2018). The tortoise or the hare: mobilization time amongst neonatal/pediatric transport teams. *Pediatrics*.

[31] Duckworth R. L. (2017). How to read and interpret end-tidal capnography waveforms. *JEMS*.

[32] De Caen A. R., Berg M. D., Chameides L. (2015). Part 12: pediatric advanced life support. *Circulation*.

[33] Hamrick J. T., Hamrick J. L., Bhalala U. (2017 Nov). End-tidal CO2-guided chest compression delivery improves survival in a neonatal asphyxial cardiac arrest model^∗^. *Pediatric Critical Care Medicine*.

[34] Sakles J. C., Chiu S., Mosier J., Walker C., Stolz U. (2013). The importance of first pass success when performing orotracheal intubation in the emergency department. *Academic Emergency Medicine*.

[35] Kim J., Kim K., Kim T. (2014). The clinical significance of a failed initial intubation attempt during emergency department resuscitation of out-of-hospital cardiac arrest patients. *Resuscitation*.

